# Supplementary material for: A vocabulary of ancient peptides at the origin of folded proteins
Source: eLife. 2015 Dec 14;4:e09410. doi: 10.7554/eLife.09410 (PMC4739770; doi:10.7554/eLife.09410)
Supplement: Figure 3—source data 1. — Multiple homologous copies of a fragment found within the same domain are indicated by a *. Fragments reported individually before are indicated by a +. DOI: http://dx.doi.org/10.7554/eLife.09410.006 [file elife-09410-fig3-data1.docx]

**Figure 3-source data 1**

Multiple sequence alignments and accession details for the 40 primordial fragments shown in Figure 3 and the 5 B-set fragments shown in Figure 3-figure supplement 1. Multiple homologous copies of a fragment found within the same domain are indicated by a *****. Fragments reported individually before are indicated by a **^+^**.

**Fragment 1^+^ (14 folds, 20 superfamilies); References: [1-5]**

hhhhhhhhcc--chhhhhhhhh

1HLV (A:28-47) a.4.1.7 KGE**I**ARR**F**N**I**--PPST**L**ST**I**LK

3L00 (A:114-135) a.4.2.0 YSH**L**AAL**AG**NpaATAA**V**KT**A**LS

1J5Y (A:27-46) a.4.5.1 GAQ**L**AEE**L**S**V**--SRQV**I**VQDIA

1P4W (A:168-187) a.4.6.2 VTE**I**AKK**L**NR--SIKT**I**SSQKK

1JHG (A:68-87) a.4.12.1 QRE**L**KNE**LGA**--GIAT**I**TR**G**SN

1KU3 (A:398-417) a.4.13.2 LEE**V**GAY**FGV**--TRER**I**RQ**I**EN

1VZ0 (A:137-156) a.4.14.1 QEE**V**ARR**VG**K--ARST**V**AN**A**LR

1R8E (A:8-27) a.6.1.3 IGE**V**SKL**A**N**V**--SIKA**L**RY**Y**DK

2R1J (A:21-40) a.35.1.2 QAA**L**GKM**VGV**--SNVA**I**SQ**W**ER

1AIS (A:1268-1287) a.74.1.2 QRE**V**AEV**A**R**V**--TEVT**V**RNRYK

1A9X (A:499-518) a.92.1.1 DAR**L**AKL**AGV**--REAE**I**RK**L**RD

2CSB (A:200-219) a.267.1.1 HDE**I**ARR**LGL**--SVSE**V**EGEKD

1F44 (A:306-326) d.163.1.1 IPE**I**MQA**GGW**t-NVNI**V**MN**F**IR

2V4J (C:66-85) d.203.1.1 VRI**L**SKNT**GF**--KLKE**V**YE**L**FP

1NR3 (A:8-27) d.236.1.1 QKK**I**ARE**L**K**T**--TRQN**V**SA**I**ER

1I3J (A:215-234) d.285.1.1 AAD**A**ARH**F**K**I**--SSGL**V**TYRVK

1GKU (B:780-799) e.10.1.1 LSD**A**NRI**L**K**F**--SVKQTMQ**I**AQ

1LDJ (A:622-641) e.40.1.1 VQQ**L**TDSTQ**I**--KMDI**L**AQ**V**LQ

4CDP (A:19-38) e.62.1.1 ARD**I**AGL**M**N**I**--REAE**L**AF**A**RV

2AVU (E:26-45) e.64.1.1 LQM**L**ESETQ**L**--SRGR**L**IK**L**YK

**Fragment 2^+^ (8 folds, 15 superfamilies); [6, 7]**

hhhhh------cccccchhhhhhhhhhh

1IXR (A:71-92)***** a.60.2.1 FEL**L**L------S**V**S**GVG**PKV**A**LA**L**LSAL

1IXR (A:106-127)***** a.60.2.1 ARL**L**T------S**A**S**GVG**RRL**A**ER**I**ALEL

1Z3E (B:282-303) a.60.3.1 EED**M**M------K**V**RN**LG**RKS**L**EE**V**KAKL

1U9L (A:389-410) a.60.4.2 MKE**L**L------E**I**E**GL**DEPT**V**EA**L**RERA

1CI4 (A:17-38) a.60.5.1 EKP**V**G------S**L**A**GIG**EVL**G**KK**L**EERG

4KLI (A:56-77) a.60.6.1 GAE**A**K------K**L**P**GVG**TKI**A**EK**I**DEFL

1UL1 (X:226-253) a.60.7.1 LCI**L**LgsdyceS**I**R**GIG**PKR**A**VD**L**IQKH

1WUD (A:574-595) a.60.8.1 ASE**M**L------S**V**N**GVG**MRK**L**ER**F**GKPF

2BCQ (A:335-356) a.60.12.1 LEL**F**S------N**I**W**GAG**TKT**A**QM**W**YQQG

1ORN (A:109-130) a.96.1.1 RDE**L**M------K**L**P**GVG**RKT**A**NV**V**VSVA

2UUB (M:16-37) a.156.1.1 DVA**L**T------Y**I**Y**GIG**KAR**A**KE**A**LEKT

2P6R (A:631-652) a.289.1.2 LLE**L**V------R**I**RH**IG**RVR**A**RK**L**YNAG

1GM5 (A:114-135) b.40.4.9 STD**I**Q------Y**A**K**GVG**PNRKKK**L**KKLG

1JX4 (A:177-198) e.8.1.7 ELD**I**A------D**V**P**GIG**NIT**A**EK**L**KKLG

3VDP (A:9-30) e.49.1.1 IEE**L**S------K**L**P**GIG**PKT**A**QR**L**AFFI

2I5H (A:129-150) e.71.1.1 MHQ**L**E------L**L**P**GVG**KKM**M**WA**I**IEER

**Fragment 3^+^ (3 folds, 3 superfamilies); [8]**

hhhhhhhhhhhhhhhcccceechhhhhhhh

1TZY (A:58-87) a.22.1.1 LTAE**IL**EL**AG**N**AA**RDNK**K**TR**I**IPRH**L**QLAI

2HUE (C:61-90) a.22.1.1 F**L**EN**VI**RD**AV**T**Y**TEHAK**R**KT**V**TAMD**V**VYAL

1B67 (A:36-65) a.22.1.2 M**G**EE**IA**SE**AV**K**LA**KHAG**R**KT**I**KAED**I**ELAR

1TAF (A:52-81) a.22.1.3 Y**V**TS**IL**DD**A**KV**YA**NHAR**K**KT**I**DLDD**V**RLAT

1JFI (A:45-74) a.22.1.3 F**L**ES**LL**KK**AC**Q**V**TQSRNAKT**M**TTSH**L**KQCI

1F6V (A:48-76) a.49.1.1 I**L**NHS**L**RL**AA**MT**A**HGKGE-R**V**NEDY**L**RQAF

1K6K (A:5-34)***** a.174.1.1 E**L**ELS**L**NM**AF**AR**A**REHRHEF**M**TVEH**L**LLAL

1K6K (A:83-112)***** a.174.1.1 S**F**QR**VL**QR**AV**FH**V**QSSG**R**NE**V**TGAN**V**LVAI

1KHY (A:9-38)***** a.174.1.1 K**F**QL**AL**AD**A**QS**LA**LGHDNQF**I**EPLH**L**MSAL

1KHY (A:86-115)*****  a.174.1.1 D**L**VR**VL**NL**C**DK**LA**QKRGDNF**I**SSEL**F**VLAA

1FNN (A:241-270) c.37.1.20 L**A**ID**IL**YRS**A**Y**AA**QQNG**R**KH**I**APED**V**RKSS

1LV7 (A:363-392) c.37.1.20 D**L**AN**LV**NE**AA**L**FA**ARGN**K**RV**V**SMVE**F**EKAK

1W5S (A:261-290) c.37.1.20 R**A**IV**AL**KM**AC**E**MA**EAMG**R**DS**L**SEDL**V**RKAV

a.49.1.1 has the same fold as the C-domain of AAA+ proteins (c.37.1.20)

**Fragment 4 (2 folds, 2 superfamilies)**

hhhhhhccchhhhhhhhhhh

1VQ8 (V:4-23) a.2.2.1 HVQ**EIRD**MTPA**E**REAE**L**DDL

2ZJR (V:2-21) a.2.2.1 KPS**EMRN**LQAT**DF**AKE**I**DAR

4V52 (DX:2-21) a.2.2.1 KAK**ELRE**KSVE**EL**NTE**L**LNL

4V4Y (B1:4-23) a.2.2.1 QLE**EAR**KLSPV**EL**EKL**V**REK

1A62 (A:2-21) a.140.3.1 NLT**ELKN**TPVS**EL**ITL**G**ENM

**Fragment 5 (2 folds, 2 superfamilies)**

hhhhhhhhccchhhhhhhhhhhhh

1EI1 (A:354-377) d.14.1.3 N**E**L**L**AE**YL**L**EN**PTD**A**KI**V**VG**KI**ID

1S16 (A:1351-1374) d.14.1.3 K**D**A**F**IL**WL**NQ**N**VQ**AA**EL**L**AEM**A**IS

1KIJ (A:354-377) d.14.1.3 Y**E**R**L**LE**IL**E**EN**PR**IA**KA**V**YE**KA**LR

1MO3 (A:303-326) d.48.1.1 K**E**N**A**RN**FL**V**EN**AD**VA**DE**I**EK**KI**KE

1U94 (A:302-325) d.48.1.1 KAN**A**TA**WL**K**DN**PET**A**KE**I**EK**KV**RE

1XP8 (A:315-338) d.48.1.1 K**E**KTIA**YI**A**E**RPE**M**EQE**I**RD**RV**MA

**Fragment 6^+^ (2 folds, 2 superfamilies); [9]**

hhhhhhhccchhhhhhhhhhc-eeeee

1TUA (A:13-38) d.51.1.1 RL**G**A**VIG**PR**GE**VKAE**I**MRR**TG**-TV**I**T**V**

1TUA (A:107-132) d.51.1.1 IK**G**R**IIG**EG**G**RAR**R**T**I**EEM**T**D-TY**I**N**V**

1ZZK (A:24-49) d.51.1.1 LA**G**S**IIGK**G**GQ**R**IK**Q**I**RHE**SG**-AS**I**K**I**

1J4W (A:13-38) d.51.1.1 AV**G**I**VIGR**N**GE**M**IK**K**I**QND**AG**-VR**I**Q**F**

2JE6 (I:163-188) d.51.1.1 KVPR**VIGK**NKSM**Y**ET**L**TSK**SG**-CS**I**F**V**

2CTE (A:27-52) d.51.1.1 HHRF**VIGK**N**GE**K**L**QD**L**ELK**TA**-TK**I**Q**I**

1WE8 (A:25-50) d.51.1.1 SV**G**R**IIGR**G**GE**T**IR**S**I**CKA**SG**-AK**I**T**C**

2ASB (A:298-323) d.52.3.1 QL**S**L**AIGK**E**GQ**N**AR**L**A**ARL**TG**-WR**I**D**I**

1WH9 (A:45-70) d.52.3.1 RTQN**VLG**EK**G**RR**IR**E**L**TAVVQ-KR**F**G**F**

1K0R (A:231-257) d.52.3.1 AK**G**A**CIG**PM**GQ**R**VR**N**V**MSEL**S**gEK**I**D**I**

**Fragment 7^+^ (6 folds, 8 superfamilies); [10-14]**

eeeechhhhhhccccccceeeee

1CZ4 (A:23-45)***** b.52.2.3 R**V**R**L**DESSRRL**L**D**A**EI**GD**V**V**E**I**E

1CZ4 (A:66-88)***** b.52.2.3 **IV**R**I**DSV**M**RNN**C**G**A**SI**GD**K**V**K**V**R

1YFB (A:17-39) b.129.1.3 R**V**V**I**PIE**L**RRT**L**G**I**AEK**D**A**L**E**I**Y

2D9R (A:78-100) b.129.2.1 **IL**G**L**RQD**I**RRA**I**GKQP**GD**S**V**Y**V**T

2VBU (A:107-129) b.43.5.2 E**I**I**A**PMK**L**REQ**F**N**L**KD**GD**V**I**K**I**L

1YLE (A:314-336) d.108.1.8 P**V**A**L**SVE**A**AEA**L**G**V**GE**G**AS**V**R**L**V

1WID (A:253-275) b.142.1.2 **L**TK**G**WSR**F**VKEKN**L**RA**GD**V**V**S**F**S

1A8P (A:19-41) b.43.4.2 **LF**S**F**KTTRNPS**L**R**F**EN**G**QF**V**M**I**G

1RQP (A:217-239) b.141.1.1 **W**TN**I**HRTDLEK**A**G**I**GY**G**AR**L**R**L**T

**Fragment 8^+^ (10 folds, 10 superfamilies); [15-18]**

ceeeeec-cchhhhhhhhhhhcccc--eeeeec

2NAC (A:192-221) c.2.1.4 MH**VG**T**VA**-**AG**R**IG**LAV**L**RR**LA**PFD**V**--H**L**H**Y**TD

1E5Q (A:4-33) c.2.1.3 KS**VLMLG**-S**G**F**V**TRPT**L**DV**L**TDS**GI**--K**V**T**VA**C

2BS2 (A:6-35) c.3.1.4 CDS**LVIG**-**GG**L**AG**LRA**A**VATQQK**GL**--STI**VL**S

1DJQ (A:390-419) c.4.1.1 DS**VLIVG**-**AG**PS**G**SEA**A**RV**LM**ES**GY**--T**V**H**L**TD

1C0P (A:1005-1034) c.4.1.2 KR**VVVLG**-S**G**V**IG**LSS**A**LI**LA**RK**GY**--S**V**H**IL**A

2JFG (A:6-35) c.5.1.1 KN**VVIIG**-**LG**LT**G**LSC**V**DF**FL**AR**GV**--TPR**VM**D

3ETJ (A:2-31) c.30.1.1 KQ**VCVLG**-N**G**Q**LG**RMLRQA**G**EPL**GI**--A**V**WP**V**G

1INL (A:92-121) c.66.1.17 KK**VLIIG**-**GG**--DGGT**L**RE**VL**KHDSveK**A**I**LC**E

1OTH (A:189-219) c.78.1.1 LT**L**S**WIG**d**G**NN**I**LHSI**M**MS**AA**KF**GM**--H**L**Q**AA**T

2V03 (A:62-91) c.79.1.1 DV**LI**E**A**T-S**G**NT**G**IAL**A**MI**AA**LK**GY**--R**M**K**LL**M

1JW9 (B:32-62) c.111.1.1 SR**VLIVG**-**LG**G**LG**CAASQY**LA**SA**GV**g-N**L**T**LL**D

1Q7E (A:10-39) c.123.1.1 IK**VL**D**F**T-**G**VQS**G**PSCTQM**LA**WF**GA**--D**V**IK**I**E

**Fragment 9 (2 folds, 2 superfamilies)**

ccceehhhhhhhcccccchhhhhhhh

1QW2 (A:88-113) d.249.1.1 K**V**VE**A**SQE**A**Q**KVGI**NP**G**DV**L**RN**V**IDK

1JX4 (A:42-67) e.8.1.7 A**V**AT**A**NYE**A**R**KFGV**KA**G**IP**I**VE**A**KKI

1T94 (A:135-160) e.8.1.7 M**L**STSNYH**A**R**RFGV**RA**A**MP**G**FI**A**KRL

1JIH (A:58-83) e.8.1.7 S**I**IA**V**SYA**A**R**KYGI**SR**M**DT**I**QE**A**LKK

**Fragment 10 (3 folds, 4 superfamilies)**

eeeeceec-ccceeecccceeee

2FF4 (A:350-371) b.26.1.2 **V**H**V**QHER**I**-RSAVT**L**ND**GD**H**I**R**I**

2AFF (A:69-90) b.26.1.2 TQ**VNG**S**VI**-DEPVR**L**KH**GD**V**I**T**I**

2G1L (A:568-589) b.26.1.2 TY**VNG**K**LV**-TEPLV**L**KS**G**NR**I**V**M**

1FM0 (D:53-74) d.15.3.1 **A**A**VN**QT**LV**-SFDHP**L**TD**GD**E**V**A**F**

1RYJ (A:45-66) d.15.3.2 **V**KK**NG**Q**IV**-IDEEE**I**FD**GD**I**I**E**V**

1TKE (A:38-59) d.15.10.1 **G**R**VNG**E**LV**-DACDL**I**ENDAQ**L**S**I**

1DM9 (A:36-57) d.66.1.3 **V**H**YNG**QRS-KPSKI**V**ELNAT**L**T**L**

1VIO (A:27-49) d.66.1.5 **V**K**ING**E**IV**kSGSVQ**I**SQE**D**E**I**Y**F**

1P9K (A:48-70) d.66.1.6 **V**K**V**D**G**A**V**EtRKRCK**I**VA**G**QT**V**S**F**

**Fragment 11 (2 folds, 2 superfamilies)**

eeeechhhcccccceecccccceeeee

1FEU (A:148-174) b.53.1.1 DS**L**H**ASDL**KLPPG**VELA**vS**P**EET**I**AA**V**

1BDF (A:108-133) d.181.1.1 GP**V**T**AADI**THDGD**VEIV**-K**P**QHV**I**CH**L**

**Fragment 12 (4 folds, 8 superfamilies)**

eeeecccceeeccceeee

1BDO (A:100-117)***** b.84.1.1 KAF**I**EV**GQ**K**V**NV**GD**T**LC**I

1BDO (A:137-154)*****  b.84.1.1 AIL**V**ES**GQ**P**V**EFD**E**P**LV**V

1DCZ (A:67-84)***** b.84.1.1 KIL**V**K**EGD**T**V**KA**GQ**T**VL**V

1DCZ (A:104-121)***** b.84.1.1 KVL**V**K**E**R**D**A**V**QG**GQ**G**LI**K

1E2W (A:213-230) b.84.2.2 DLI**V**K**EGQ**T**V**QAD**Q**P**L**TN

1GPR (A:97-114) b.84.3.1 TSF**V**S**EGD**R**V**EP**GQ**K**LL**E

1V8Q (A:22-39) b.84.4.1 GVKRY**EGQ**V**V**RA**G**NI**LV**R

1O4U (A:62-79) d.41.2.1 KFN**V**E**DGE**Y**L**EGTGV**IG**E

1UOU (A:422-439) d.41.3.1 ELL**V**DV**GQ**R**L**RR**G**TP**WL**R

4EAD (A:384-401) d.41.3.1 TDM**A**RL**GD**Q**V**DGQRP**LA**V

4G7H (D:1310-1327) e.29.1.2 RLL**V**K**DGD**Y**V**EA**GQ**P**L**TR

1VF7 (A:53-70) f.46.1.1 KRL**F**K**EG**SD**V**KA**GQ**Q**LY**Q

**Fragment 13^+^ (5 folds, 30 superfamilies); [19]**

ceeeeeeccccceeeeeecc--eeeeeecccc-----ceeeeeec

1GEN (A:567-604) b.66.1.1 R**V**D**AAF**NWSKNKKT**YI**FAG**D**--**KF**WR**YN**EVKK-----KMDPGFPK

1TL2 (A:42-83) b.67.1.1 N**F**K**FLFL**SPGG-E**LYG**VLN**D**--**KI**YK**G**TPPTHdndnwMGRAKK**I**G

1CRU (A:28-64) b.68.2.1 KPH**ALLW**GPD-NQ**IWL**TERAtG**KI**LR**VN**PES------GSV-KT**V**F

1TBG (A:57-94)***** b.69.4.1 K**I**Y**AM**H**W**GTDSRL**LV**SASQ**D**-G**KL**II**WD**SYT------TNKVHA**I**P

1TBG (A:272-309)***** b.69.4.1 G**I**TS**V**S**F**SKSGRL**LLA**GYD**D**-FNCNV**WD**ALK------ADRAGV**L**A

1HZU (A:180-219) b.70.2.1 A**V**H**I**SR**M**SASGRY**LLV**IGR**D**-A**RI**DM**ID**LWAKE----PTKVAE**I**K

**Fragment 14 (2 folds, 2 superfamilies)**

eecccccceecchhhhhhhhhhh

2PQ8 (A:208-230) d.108.1.1 **W**L**C**EY**CLK**Y**M**KYEKS**Y**RF**HL**GQC

1M36 (A:8-30) g.37.1.2 **Y**L**C**EF**CLK**Y**M**KSRTI**L**QQ**HM**KKC

1X6H (A:48-70) g.37.1.1 **F**V**C**SK**CGK**T**F**TRRNT**M**AR**HA**DNC

2DRP (A:141-163) g.37.1.1 **Y**P**C**PF**CFK**E**F**TRKDN**M**TA**HV**KII

**Fragment 15 (5 folds, 7 superfamilies)**

eeeeccccceeeeee

3LGI (A:203-217) b.47.1.1 GA**LV**NSL**G**E**LMGI**NT

1AGJ (A:197-211) b.47.1.1 SG**IF**NSN**G**E**LVGI**HS

1TIF (A:16-30) d.15.8.1 VR**LI**DQN**G**DQ**LGI**KS

2NYC (A:223-237)***** d.37.1.1 VP**II**DEN**G**Y**LI**N**V**YE

2NYC (A:295-309)***** d.37.1.1 FF**VV**DDV**GRLVGV**LT

1F5M (A:135-149) d.110.2.1 VP**II**SND**GK**T**LGV**ID

1N9L (A:104-118) d.110.3.6 TP**I**KTPD**GRVS**K**F**VG

1P0Z (A:110-124) d.110.6.1 SP**I**QDAT**GKVIGI**VS

1U7Q (A:430-444) d.220.1.1 PL**VV**VEGS**RVLGV**IA

**Fragment 16^+^ (3 folds, 3 superfamilies); [20, 21]**

ceeeeecccccchhhhhhhhhhh

1GSI (A:1-23) c.37.1.1 M**LIAI**E**G**VDGA**GK**RTL**V**EK**L**SGA

1M7G (A:26-48) c.37.1.4 LT**IWLTG**LSAS**GKS**TL**A**VE**L**EHQ

1RZ3 (A:23-45) c.37.1.6 L**VLGI**D**G**LSRS**GKT**TL**A**NQ**L**SQT

1UJ2 (A:21-43) c.37.1.6 F**LIGVSG**GTAS**GKS**SV**C**AK**I**VQL

2QM8 (A:56-78) c.37.1.10 IR**VGITG**VPGV**GKS**TT**I**DA**L**GSL

1KNQ (A:9-31) c.37.1.17 H**IYVL**M**G**VSGS**GKS**AV**A**SE**V**AHQ

2JFG (A:105-125) c.72.2.1 P**IVAITG**SN--**GKS**TVTTL**V**GEM

2GC6 (A:40-60) c.72.2.2 R**YI**H**VTG**TN--**GKG**SA**A**NA**I**AHV

1KO7 (A:145-167) c.91.1.2 V**GVLITG**DSGI**GKS**ET**A**LE**L**IKR

1KNX (A:148-170) c.91.1.2 V**GVLLTG**RSGI**GKS**EC**A**LD**L**INK

**Fragment 17^+^ (3 folds, 3 superfamilies); [22]**

hhhhccccc

1I8O (A:10-18) a.3.1.1 **F**KQ**CM**T**CH**R

1JDL (A:12-20) a.3.1.1 **F**KK**CM**A**CH**R

1C52 (A:8-16) a.3.1.1 **Y**AQ**CA**G**CH**Q

2YKZ (A:113-121) a.24.3.2 **G**AS**C**KA**CH**D

1S05 (A:114-122) a.24.3.2 **G**KA**CG**N**CH**E

1SP3 (A:19-27) a.138.1.3 TTQ**CL**T**CH**E

1H21 (A:206-214) a.138.1.1 **V**SS**CG**E**CH**M

1FGJ (A:76-84)***** a.138.1.3 RKD**CV**E**CH**S

1FGJ (A:142-150)***** a.138.1.3 EVG**CI**D**CH**V

1FGJ (A:169-177)***** a.138.1.3 **A**DT**CG**T**CH**L

**Fragment 18^+^ (2 folds, 2 superfamilies); [23]**

cccccchhhhhhhcc

2BS2 (B:149-163)***** a.1.2.1 DR**CI**E**CG**C**CI**A**ACG**T

2BS2 (B:206-220)***** a.1.2.1 FG**CM**TL**L**A**C**HD**VCP**K

2WDQ (B:147-161)***** a.1.2.1 YE**CI**L**CA**C**C**STS**CP**S

2WDQ (B:204-218)***** a.1.2.1 FR**C**HSI**M**N**CV**S**VCP**K

2FDN (A:6-20)***** d.58.1.1 EA**CI**S**CG**A**C**EPE**CP**V

2FDN (A:35-49)***** d.58.1.1 DT**CI**D**CG**A**CA**G**VCP**V

1XER (A:43-57) d.58.1.3 DL**CI**AD**G**S**CI**N**ACP**V

2C42 (A:687-701)***** d.58.1.5 EN**CI**Q**C**NQ**CA**F**VCP**H

2C42 (A:743-757)***** d.58.1.5 LD**CM**G**CG**N**CA**D**ICP**P

3C8Y (A:145-159) d.58.1.5 TK**CL**L**CG**R**CV**N**AC**GK

1SJ1 (A:9-23) d.58.1.4 DT**CI**GD**A**I**CA**S**LCP**D

**Fragment 19^+^ (3 folds, 3 superfamilies); [24]**

eeeceeeeccccccceeeeccceeeehhhhh

1VYX (A:8-38) g.44.1.3 V**C**WI**C**NEE**LG**NERFRA**C**G**C**TGELEN**V**HRS**C**L

1G25 (A:5-35) g.44.1.1 G**C**PR**C**KTTK**Y**RNPSLK**L**MVNV**C**GHT**L**CES**C**V

3UEJ (A:243-273) g.49.1.1 F**C**DH**C**GSL**LW**GLVKQG**L**K**C**ED**C**GMN**V**HHK**C**R

1ZBD (B:93-123) g.50.1.1 R**C**IL**C**GEQ**LG**MLGSAS**V**V**C**ED**C**KKN**V**CTK**C**G

**Fragment** **20** **(2 folds, 2 superfamilies)**

cccccccccccceee

1EP3 (B:223-237) c.25.1.3 R**M**A**C**G**IGAC**YA**CV**EH

2BS2 (B:54-68) d.15.4.2 D**F**V**C**R**AGIC**GS**CG**MM

3WCQ (A:37-51) d.15.4.1 P**Y**S**C**R**AGAC**ST**CA**GK

1DOI (A:60-74) d.15.4.1 P**F**S**C**R**AGAC**AN**CA**AI

**Fragment 21 (2 folds, 2 superfamilies)**

ccccccccchhhhhhhhhhh

2SCP (B:104-123)***** a.39.1.5 **D**T**N**E**D**NN**I**SRD**EYGIFF**GM**L**

2SCP (B:138-157)***** a.39.1.5 **D**T**N**N**DG**L**L**SLE**EFVIAG**SD**F**

1EG3 (A:187-206) a.39.1.7 **D**TGRT**G**R**I**RVLS**F**KT**GI**IS**L**

2Y3N (B:8-27)***** a.139.1.0 **D**L**N**G**DG**V**I**NMA**DVMILA**QS**F**

2Y3N (B:41-60)***** a.139.1.0 **D**L**N**N**DG**V**I**NSD**DAIILA**QY**F**

**Fragment 22 (2 folds, 2 superfamilies)**

hhhhhhhhhhhhhhccccchhhhhhhhhhhhhhhh

2FZF (A:32-66)***** a.25.1.1 EI**GA**RE**FY**KS**L**AEK**I**KIEAL**K**EK**I**NWL**A**EEEKKHE

2FZF (A:117-151)***** a.25.1.1 EE**IA**AE**FY**LK**L**EEM**V**KEEEK**K**RL**M**RYL**A**DMERGHY

1Z72 (A:58-92) a.132.1.3 FD**AF**LS**ML**GA**C**VAH**A**DKLES**K**LR**F**AKQ**L**GFLEADE

**Fragment 23 (2 folds, 2 superfamilies)**

hhhhhhhhccceeeeecc

1ECS (A:16-33) d.32.1.1 ST**A**A**FYERLGF**GI**VF**RDA

1CJX (A:25-42) d.32.1.3 TLEP**IFE**I**MGF**TK**VA**THR

1SS4 (A:21-38) d.32.1.6 NA**I**S**FFE**E**IGL**NLE**G**R**A**N

2GAN (A:152-169) d.108.1.1 AYSY**YY**M**K**K**GF**RE**IM**R**Y**K

1GHE (A:136-153) d.108.1.1 VAEA**FY**SA**LAY**TR**VG**E**L**P

1N71 (A:142-159) d.108.1.1 HP**Y**E**FYEKLGY**KI**VG**V**L**P

1VHS (A:126-143) d.108.1.1 PS**L**K**LFEK**H**GF**AE**WG**L**F**P

**Fragment 24^+^ (2 folds, 2 superfamilies); [25]**

hhhhhhhhhhhhccchhhhhhhhhh

1XB2 (B:59-83) a.5.2.2 SKE**LL**MK**LR**RK**TGYSF**I**N**C**K**K**AL**ET

1AIP (C:3-27) a.5.2.2 Q**M**E**LIK**K**LR**EA**TGAGM**M**D**V**K**R**AL**ED

1EFU (B:4-28) a.5.2.2 T**A**S**LVK**E**LR**ER**TGAGM**M**D**C**K**K**AL**TE

1CTF (A:65-89) d.45.1.1 K**V**A**VIK**A**VR**GA**TGLGL**K**E**A**K**D**LV**ES

1DD3 (A:70-94) d.45.1.1 K**I**Q**VIK**V**VR**EI**TGLGL**K**E**A**K**D**LV**EK

**Fragment 25^+^ (2 folds, 7 superfamilies); [26]**

eeeeeeeee-cceeeeeeee

1QJP (A:124-142) f.4.1.1 A**GGV**E**Y**A**I**T-PE**I**ATR**L**E**Y**Q

1QD6 (A:196-213) f.4.2.1 Q**L**K**I**G**Y**H**L**G--D**A**V**L**S**A**K**G**Q

1T16 (A:81-99)***** f.4.3.4 N**M**H**F**V**A**P**I**N-DQ**F**G**W**G**A**S**I**T

1T16 (A:129-147)***** f.4.3.4 N**L**S**G**A**Y**R**L**N-NA**W**S**F**G**L**G**F**N

1T16 (A:210-228)***** f.4.3.4 N**AGI**L**Y**E**L**D-KNNR**Y**A**L**T**Y**R

1T16 (A:276-294)***** f.4.3.4 E**V**S**G**YNR**V**D-PQ**W**A**I**H**Y**S**L**A

1T16 (A:328-346)***** f.4.3.4 A**LG**TT**Y**Y**Y**D-DN**W**T**F**RTG**I**A

1T16 (A:370-388)***** f.4.3.4 S**AG**TT**Y**A**F**N-KD**A**S**V**D**V**G**V**S

1I78 (A:234-252) f.4.4.1 A**V**N**A**G**Y**Y**V**T-PN**A**K**V**Y**V**E**G**A

1UYN (A:886-904) f.4.5.1 F**AGI**RHD**A**G-DI**G**Y**L**K**G**L**F**S

1TLY (A:217-234) f.4.6.1 SH**IL**ALN**Y**--DH**W**H**Y**S**V**V**A**R

2GR8 (A:1062-1081) d.24.1.4 A**IGV**SRISDnGK**V**I**I**R**L**S**G**T

**Fragment 26 (3 folds, 3 superfamilies)**

eeecccceeeccceeeec

1IJQ (A:675-692) g.3.11.1 **C**A**C**PD**GMLL**AR**D**MRS**CL**T

2Y5F (L:109-126) g.3.11.1 **C**S**C**AR**GY**T**L**AD**N**GKA**CI**P

1NT0 (A:146-163) g.3.11.1 **C**S**C**RV**GYIL**HQ**N**KHT**C**SA

1CCV (A:36-52) g.22.1.1 **C**Q**C**QE**GFL**RNG**E**G-A**CV**L

2H9E (C:50-66) g.22.1.1 **C**V**C**EE**GFY**RNK**D**D-K**CV**S

1D4V (A:79-96) g.24.1.1 GL**C**PP**G**HH**I**SE**D**GRD**CI**S

1EXT (A:137-152) g.24.1.1 **C**T**C**HA**GFFL**RE**N**--E**CV**S

**Fragment 27^+^ (2 folds, 2 superfamilies); [27]**

hhhhhhhhhhhhh--chhhhhcchhhhhhhhh

2C9W (A:163-192) a.271.1.1 **L**QHL**C**RLT**I**NKCT--GA**I**WG**L**P**L**PTR**L**K**D**Y**LE**

1LM8 (V:158-189) b.3.3.1 **L**KER**C**LQV**V**RSLVKPEN**Y**RR**L**D**I**VRS**L**Y**E**D**LE**

**Fragment 28^+^ (7 folds, 12 superfamilies); [28]**

hhhhhhhhhhhhhhccchhhhhhhhhhhhhhh

1WR0 (A:14-45) a.7.14.1 K**A**ID**L**ASK**A**AQEDKA**G**N**YEEAL**QL**Y**QH**AV**QYF

2CRB (A:14-45) a.7.16.1 L**A**HQQSRR**A**DR**LL**AA**G**K**YEEAI**SCHRK**A**TTYL

1OM2 (A:19-50) a.23.4.1 F**F**LEEIQL**G**EE**LL**AQ**GDYE**K**GV**DH**L**TN**AI**AVC

1UG7 (A:18-49) a.24.24.1 R**W**GASLRR**G**AD**F**DSW**G**Q**L**V**EAI**DE**Y**QI**LA**RHL

1QSA (A:412-443) a.118.5.1 SKTEQAQL**A**RY**AF**NNQW**WD**LS**V**QATIA**G**KLWD

2O02 (A:2-33) a.118.7.1 DKNE**L**VQKAKL**A**EQAER**YDDMA**AC**M**KS**V**TEQG

1ELW (A:3-34)***** a.118.8.1 Q**V**NE**L**KEK**G**NK**AL**SV**G**N**IDDAL**QC**Y**SE**AI**KLD

1ELW (A:37-68)***** a.118.8.1 NHVL**Y**SNR**S**AA**YA**KK**GDY**QK**AY**ED**G**CKT**V**DLK

1ELW (A:71-102)***** a.118.8.1 W**G**KG**Y**SRK**A**AA**L**EFLNR**FEEA**KRT**Y**EE**GL**KHE

1WY6 (A:125-156) a.118.20.1 S**A**SI**L**VAI**A**NA**L**RRV**GD**ER**DA**TTL**L**IE**AC**KKG

2A9U (A:39-70) a.118.23.1 S**A**LK**I**FKT**A**EE**C**RLDR**D**E**E**R**AY**VL**Y**MK**YV**TVY

2IJQ (A:29-60) a.246.2.1 T**L**RR**A**VVH**G**VR**LY**NS**GEF**H**E**SHDC**F**EDE**W**YNY

2CFU (A:448-479) d.157.1.13 G**A**ER**L**LEQ**A**RAS**Y**AR**GEY**RW**VV**EV**V**NR**LV**FAE

1ZBP (A:30-61) e.61.1.1 D**A**SLRSSF**I**EL**LC**ID**GDFE**R**A**DEQ**L**MQS**I**KLF

**Fragment 29 (2 folds, 2 superfamilies)**

hhhhhhhhhhhhhhccccchhhhhhhhh

3A8G (B:32-59)***** b.34.4.4 **W**EHLP**Y**S**L**MF**AG**VAeL**GAFS**V**D**E**V**RY**VV**

3A8G (B:73-99)***** b.34.4.4 **Y**ERY**VI**G**V**AT**LM**VE-**KGILT**Q**D**E**L**ES**LA**

3A8G (A:18-44) d.149.1.1 **V**SDR**AW**A**L**FR**AL**DG-**KGLV**PDGY**V**EG**W**K

1V29 (A:29-55) d.149.1.1 **W**EAR**A**KA**L**ES**LL**IE-**K**R**LLS**S**D**A**I**ER**VI**

**Fragment 30 (2 folds, 2 superfamilies)**

hhhhhhhhhhhhhhhccchhhhhhhhhhhhh

3BHP (A:6-36) a.2.21.1 KI**AR**IN**ELAAK**A**K**AG**VIT**E**EEKAE**QQK**L**R**QE**

1T3W (A:547-577) a.236.1.1 LE**LR**QE**ELIAR**E**R**TH**GLS**N**EERLE**LWT**L**N**QE**

**Fragment 31 (2 folds, 2 superfamilies)**

chhhhhhhhhhhccccchhhhhhh

3BUL (A:658-681) a.46.1.1 **E**VNKR**L**EYS**LV**K**G**IT**E**F**I**EQDTEE

1JR3 (A:247-270) a.80.1.1 **D**QALS**L**VEA**MV**E**A**NG**E**R**V**MALINE

1JR3 (D:212-235) a.80.1.1 FTPFH**W**VDA**LL**M**G**KSKR**A**LHILQQ

1SXJ (D:266-289) a.80.1.1 **D**ILIE**I**VEK**V**KS**G**DF**D**E**I**KKYVNT

**Fragment 32 (2 folds, 2 superfamilies)**

hhhhhhhhhhhhhhhhcccchhhceeeeee

1HUX (A:35-64) c.55.1.5 GTGTS**G**P**A**RS**I**SE**VL**E**N**AH**M**KKE**DM**A**F**TL**A**

1NBW (A:43-72) c.55.1.6 RDNIA**G**T**L**A**AL**EQ**AL**AKTP**W**SMS**DV**SR**I**Y**L**

2D0O (A:44-73) c.55.1.6 LRNVF**GI**QE**AL**AL**VA**RGAG**I**AVS**DI**S**LI**R**I**

2A6A (A:31-60) c.55.1.9 KKHAE**IL**PV**VV**KK**LL**D**E**LD**L**KVK**DL**D**VV**G**V**

1OX0 (A:273-302) c.95.1.1 HPEGQ**GAI**K**AI**KL**AL**E**E**AE**I**SPEQ**V**A**YV**N**A**

1TQY (A:279-308) c.95.1.1 KADGRE**MA**ET**I**RV**AL**D**E**SRTDAT**DI**D**YI**N**A**

1TED (A:283-312) c.95.1.2 GYIFS**GVA**P**VV**TE**ML**W**D**NG**L**QIS**DI**D**LW**A**I**

**Fragment** **33** **(2 folds, 2 superfamilies)**

eeeeeecccehhhhhhhhhhhcc

2C60 (A:56-78) d.15.2.2 R**II**A**F**SRP**V**K**Y**ED**V**EHK**V**TT**VF**G

1WMH (A:28-50) d.15.2.2 M**I**TH**F**EPS**I**S**F**EG**L**CNE**V**RD**MC**S

1OEY (A:362-384) d.15.2.2 V**VM**KTQPG**L**P**Y**SQ**V**RDM**V**SKK**L**E

1IP9 (A:24-46) d.15.2.2 F**AL**M**L**KGDTT**Y**KE**L**RSK**I**APR**I**D

2I1S (A:24-46) d.343.1.1 RR**I**Q**V**PEN**Y**T**F**LD**L**HKA**I**QA**VM**D

**Fragment 34^+^ (3 folds, 4 superfamilies); [23, 29]**

eeeeeecccccchhhhhhhhhhhhhh

1D1Q (A:7-31) c.44.1.1 IS**VAF**IAL-**G**NFCR**S**P**MA**EA**IF**KHE**V**

1JF8 (A:4-28) c.44.1.1 KT**IYF**IST-**G**NSAR**S**Q**MA**EG**WG**KEI**L**

1VKR (A:378-402) c.44.2.1 RK**IIV**ACD-AGMGS**SAMG**AG**VL**RKK**I**

2R4Q (A:172-196 c.44.2.2 IL**AV**TACP-TGIAH**TFMA**AD**AL**KEK**A**

1MKP (A:287-311) c.45.1.1 CG**VLV**HSLA**G**I-SR**SV**T**V**TV**AY**LMQK

1OHE (A:308-332) c.45.1.1 GA**IAV**HSKA**G**L-GR**TG**T**L**IA**CY**IMKH

1YT8 (A:187-210) c.46.1.2 TR**VIV**NCA-**G**R-TR**SIIG**TQS**L**LNA**G**

1TQ1 (A:83-106) c.46.1.3 DN**IIV**GCQ-**S**G-GR**SI**K**A**TTD**L**LHA**G**

**Fragment 35 (2 folds, 2 superfamilies)**

hhhhhhhhhhhcccccceeeecceeeeec

1IN0 (A:106-134) d.58.49.1 MAKK**I**TK**LVK**D**S**K**I**K**V**QTQIQ**G**EQ**VRV**T**G**

1WIH (A:46-74) d.67.3.1 CTAA**A**IK**AIR**E**S**G**M**N**L**NPEVE**G**TL**IRV**P**I**

1WQG (A:74-102) d.67.3.1 QLRA**I**ET**AIR**N**S**D**L**G**V**NPTND**G**AL**IRV**A**V**

**Fragment 36 (2 folds, 2 superfamilies)**

ceeeeccchhhhhhhhhhhcceeeec

1O13 (A:66-91) c.55.5.1 **ELVI**VRG**IG**RRA**I**AA**F**EA**MGV**KV**I**KG

1EO1 (A:67-92) c.55.5.1 K**AVI**ASSP**G**PNA**F**EV**L**NE**LGI**KI**Y**RA

1P90 (A:167-192) c.55.5.2 Q**VLY**V**V**S**IG**GPA**A**AK**V**VR**AGI**HP**L**KK

1NMO (A:188-213) c.135.1.1 **DAFI**T**G**E**V**SEQT**I**HS**A**REQ**GL**HF**Y**AA

2GX8 (A:306-331) c.135.1.1 **DVYV**T**G**D**MY**YHV**A**HD**A**MM**LGL**NI**V**DP

2FYW (A:198-223) c.135.1.1 **DVYI**T**G**D**IY**YHT**A**QD**M**LSD**GL**LA**L**DP

**Fragment 37 (2 folds, 2 superfamilies)**

bbbbbcchhhhhhhh

1MLA (A:87-101) c.19.1.1 **MMAG**H**SLG**E**Y**S**ALVC**

1OXW (A:72-86) c.19.1.3 **VIGG**T**S**T**GGL**LT**AMI**

1JJF (A:167-181) c.69.1.2 **AIAG**L**SMGGG**QS**F**N**I**

3C8D (A:276-290) c.69.1.2 **VVAG**Q**SFGGL**S**ALYA**

2PBL (A:131-145) c.69.1.2 **VLAG**H**SAGG**HL**VA**R**M**

1MTZ (A:100-114) c.69.1.7 **FLMG**S**SYGGA**L**ALAY**

**Fragment 38 (2 folds, 2 superfamilies)**

hhhhhhhhhhhhhhccchhhhhhhhhhhhh

2AHO (B:101-130) a.60.14.1 QR**L**DK**IL**EL**VS**Q**K**LKLSEK**DAW**EQ**VA**WKLE

2A19 (A:105-134) a.60.14.1 KT**V**HS**IL**RY**CA**E**K**FQIPLE**ELY**KT**IA**WPLS

3SJ5 (A:46-75) d.278.1.1 DE**V**RR**IF**AK**VS**E**K**TGKNVN**EIW**RE**VG**RQNI

**Fragment 39 (2 folds, 2 superfamilies)**

hhhhhhhhhhhhhhhc--ccchhhhhhhhhhhhhh

1S5Q (B:303-335) a.59.1.1 E**F**NH**AI**NY**V**NK**IK**NR**F**--QGQPDI**Y**KA**FL**E**IL**HTY

2CR7 (A:9-41) a.59.1.1 H**V**ED**AL**TY**L**DQ**VK**IR**F**--GSDPAT**Y**NG**FL**E**IM**KEF

1Q2Z (A:42-76) a.118.19.1 Y**F**MK**SI**DC**I**RA**FR**EE**A**ikFSEEQR**F**NN**FL**K**AL**QEK

**Fragment 40 (2 folds, 2 superfamilies)**

cccccccchhhhhhhhhhh

1LBU (A:36-54)***** a.20.1.1 **L**A**IDG**Q**FG**P**AT**KA**AV**QR**F**Q

1LBU (A:59-77)***** a.20.1.1 **L**A**ADGIAG**P**ATF**NK**I**YQ**L**Q

2IKB (A:103-121) d.2.1.9 **V**PD**DGVIG**A**VSL**K**AI**NS**L**P

2NR7 (A:112-130) d.2.1.9 **V**Q**ADGIVG**NK**TL**Q**AV**NS**A**D

**B-Set**

**Fragment B1^+^ (6 folds, 6 superfamilies); [30]**

eeeeecccceeeee

1QBA (A:842-855) b.1.18.2 IEY**S**T**D**G**G**KQ**W**QR**Y**

1D7P (M:2262-2274) b.18.1.2 ISS**S**Q**D**-**G**HQ**W**TL**F**

1VKD (A:73-85)***** b.67.2.4 F**G**R**S**K**D**-**G**IN**W**EIE

1VKD (A:131-143)***** b.67.2.4 V**G**M**T**K**D**-**F**KT**F**VR**L**

1VKD (A:184-196)***** b.67.2.4 LSE**S**P**D**-**M**IH**W**GNH

3SIL (A:70-83)***** b.68.1.1 A**A**R**S**T**D**G**G**KT**W**NKK

3SIL (A:253-266)*****  b.68.1.1 S**F**E**T**K**D**F**G**KT**W**TE**F**

1SQJ (A:194-207) b.69.13.1 M**Y**V**T**H**D**G**G**VS**W**EP**V**

1LNI (A:79-92) d.1.1.2 D**Y**Y**T**G**D**H**Y**AT**F**SL**I**

**Fragment B2 (2 folds, 2 superfamilies)**

hhhhhhhhhhhhhhhhhhhhhccchhhhhhhhhhhhhhhhhhhhhhh

1VH6 (A:22-68) a.24.19.1 EL**T**L**MLY**N**GCL**KF**IRLAA**QA**I**EN**DDM**ERKNEN**LIK**AQN**II**QE**L**NFT**L**

2NW8 (A:56-102) a.266.1.1 QT**S**E**LWL**K**LLA**HE**LRAAI**VH**L**QR**DEV**WQCRKV**LAR**SKQ**VL**RQ**L**TEQ**W**

**Fragment B3 (2 folds, 2 superfamilies)**

ceeeeeecceeeeeeec

1YRR (A:18-34) b.92.1.5 **D**H**AVVI**ADGL**I**K**SV**CP**V**

2OOD (A:35-51) b.92.1.4 **D**G**LMVV**TDGV**I**K**AF**GP**Y**

2FB5 (A:135-151) d.320.1.1 **D**G**AVLV**KNNH**I**V**SA**AN**I**

**Fragment B4 (2 folds, 2 superfamilies)**

cchhhhhhhhhhccceeeeeecceeeeecc

1WHZ (A:5-34) d.50.3.2 P**RPEEVARKL**R**RLGF**VER**MA**KGGHR**LY**THP

2G7J (A:3-32) d.198.5.1 L**RPDEVARVL**E**KAGF**TVD**VV**TNKTY**GY**RRG

**Fragment B5 (2 folds, 2 superfamilies)**

hhhhhhhhhhhhhhhhcchhhhhhhhhhhhhc

2P06 (A:42-73) a.204.1.3 SR**L**F**EEM**DE**L**REA**V**EKE**DW**EN**L**RDE**L**LD**V**AN**F**

1VMG (A:30-61) a.204.1.2 TW**L**V**EEV**GE**L**AEA**L**LSN**NL**DS**I**QEE**L**AD**V**IA**W**

1SR2 (A:816-847) a.24.10.2 DT**V**P**DDV**KR**L**YTE**A**ATS**DF**AA**L**AQT**A**HR**L**KG**V**

**References**

1. Aravind, L., et al., *The many faces of the helix-turn-helix domain: transcription regulation and beyond.* FEMS Microbiol Rev, 2005. **29**(2): p. 231-62.

2. Brennan, R.G. and B.W. Matthews, *The helix-turn-helix DNA binding motif.* J Biol Chem, 1989. **264**(4): p. 1903-6.

3. Pabo, C.O. and R.T. Sauer, *Protein-DNA recognition.* Annu Rev Biochem, 1984. **53**: p. 293-321.

4. Sauer, R.T., et al., *Homology among DNA-binding proteins suggests use of a conserved super-secondary structure.* Nature, 1982. **298**(5873): p. 447-51.

5. Suzuki, M. and S.E. Brenner, *Classification of multi-helical DNA-binding domains and application to predict the DBD structures of sigma factor, LysR, OmpR/PhoB, CENP-B, Rapl, and Xy1S/Ada/AraC.* FEBS Lett, 1995. **372**(2-3): p. 215-21.

6. Doherty, A.J., L.C. Serpell, and C.P. Ponting, *The helix-hairpin-helix DNA-binding motif: a structural basis for non-sequence-specific recognition of DNA.* Nucleic Acids Res, 1996. **24**(13): p. 2488-97.

7. Shao, X. and N.V. Grishin, *Common fold in helix-hairpin-helix proteins.* Nucleic Acids Res, 2000. **28**(14): p. 2643-50.

8. Alva, V., et al., *On the origin of the histone fold.* BMC Struct Biol, 2007. **7**: p. 17.

9. Grishin, N.V., *KH domain: one motif, two folds.* Nucleic Acids Res, 2001. **29**(3): p. 638-43.

10. Alva, V., et al., *Cradle-loop barrels and the concept of metafolds in protein classification by natural descent.* Curr Opin Struct Biol, 2008. **18**(3): p. 358-65.

11. Ammelburg, M., et al., *A CTP-dependent archaeal riboflavin kinase forms a bridge in the evolution of cradle-loop barrels.* Structure, 2007. **15**(12): p. 1577-90.

12. Coles, M., et al., *The solution structure of VAT-N reveals a 'missing link' in the evolution of complex enzymes from a simple betaalphabetabeta element.* Curr Biol, 1999. **9**(20): p. 1158-68.

13. Coles, M., et al., *AbrB-like transcription factors assume a swapped hairpin fold that is evolutionarily related to double-psi beta barrels.* Structure, 2005. **13**(6): p. 919-28.

14. Coles, M., et al., *Common evolutionary origin of swapped-hairpin and double-psi beta barrels.* Structure, 2006. **14**(10): p. 1489-98.

15. Buehner, M., et al., *D-glyceraldehyde-3-phosphate dehydrogenase: three-dimensional structure and evolutionary significance.* Proc Natl Acad Sci U S A, 1973. **70**(11): p. 3052-4.

16. Dym, O. and D. Eisenberg, *Sequence-structure analysis of FAD-containing proteins.* Protein Sci, 2001. **10**(9): p. 1712-28.

17. Rossmann, M.G., D. Moras, and K.W. Olsen, *Chemical and biological evolution of nucleotide-binding protein.* Nature, 1974. **250**(463): p. 194-9.

18. Wierenga, R.K., P. Terpstra, and W.G. Hol, *Prediction of the occurrence of the ADP-binding beta alpha beta-fold in proteins, using an amino acid sequence fingerprint.* J Mol Biol, 1986. **187**(1): p. 101-7.

19. Chaudhuri, I., J. Soding, and A.N. Lupas, *Evolution of the beta-propeller fold.* Proteins, 2008. **71**(2): p. 795-803.

20. Matte, A., et al., *Crystal structure of Escherichia coli phosphoenolpyruvate carboxykinase: a new structural family with the P-loop nucleoside triphosphate hydrolase fold.* J Mol Biol, 1996. **256**(1): p. 126-43.

21. Walker, J.E., et al., *Distantly related sequences in the alpha- and beta-subunits of ATP synthase, myosin, kinases and other ATP-requiring enzymes and a common nucleotide binding fold.* EMBO J, 1982. **1**(8): p. 945-51.

22. Mathews, F.S., *The structure, function and evolution of cytochromes.* Prog Biophys Mol Biol, 1985. **45**(1): p. 1-56.

23. Lupas, A.N., C.P. Ponting, and R.B. Russell, *On the evolution of protein folds: are similar motifs in different protein folds the result of convergence, insertion, or relics of an ancient peptide world?* J Struct Biol, 2001. **134**(2-3): p. 191-203.

24. Grishin, N.V., *Treble clef finger--a functionally diverse zinc-binding structural motif.* Nucleic Acids Res, 2001. **29**(8): p. 1703-14.

25. Wieden, H.J., W. Wintermeyer, and M.V. Rodnina, *A common structural motif in elongation factor Ts and ribosomal protein L7/12 may be involved in the interaction with elongation factor Tu.* J Mol Evol, 2001. **52**(2): p. 129-36.

26. Remmert, M., et al., *Evolution of outer membrane beta-barrels from an ancestral beta beta hairpin.* Mol Biol Evol, 2010. **27**(6): p. 1348-58.

27. Zhang, J.G., et al., *The conserved SOCS box motif in suppressors of cytokine signaling binds to elongins B and C and may couple bound proteins to proteasomal degradation.* Proc Natl Acad Sci U S A, 1999. **96**(5): p. 2071-6.

28. D'Andrea, L.D. and L. Regan, *TPR proteins: the versatile helix.* Trends Biochem Sci, 2003. **28**(12): p. 655-62.

29. Fauman, E.B., et al., *Crystal structure of the catalytic domain of the human cell cycle control phosphatase, Cdc25A.* Cell, 1998. **93**(4): p. 617-25.

30. Copley, R.R., R.B. Russell, and C.P. Ponting, *Sialidase-like Asp-boxes: sequence-similar structures within different protein folds.* Protein Sci, 2001. **10**(2): p. 285-92.
